# Evidence for ethanol-dependent acetic acid resistance in *Acetobacter pasteurianus* strain SKU1108

**DOI:** 10.1128/aem.02034-25

**Published:** 2025-11-26

**Authors:** Akari Narimatsu, Kaho Murakami, Naoya Kataoka, Riku Yamashita, Kazunobu Matsushita, Minenosuke Matsutani, Uraiwan Tippayasak, Gunjana Theeragool, Toshiharu Yakushi

**Affiliations:** 1 Joint Degree Program of Kasetsart University and Yamaguchi University, Graduate School of Science and Technology for Innovation, Yamaguchi University 98402 , Yamaguchi, Japan; 2 Division of Agricultural Science, Graduate School of Science and Technology for Innovation, Yamaguchi University 13150, Yamaguchi, Japan; 3 Department of Biological Chemistry, Faculty of Agriculture, Yamaguchi University 13150, Yamaguchi, Japan; 4 Research Center for Thermotolerant Microbial Resources, Yamaguchi University 13150, Yamaguchi, Japan; 5 Department of Food, Aroma and Cosmetic Chemistry, Faculty of Bioindustry, Tokyo University of Agriculture 13126, Abashiri, Hokkaido, Japan; 6 Department of Microbiology, Faculty of Science, Kasetsart University 54775, Bangkok, Thailand; Shanghai Jiao Tong University, Shanghai, China

**Keywords:** acetic acid bacteria, acetic acid resistance, *Acetobacter*, protonophore

## Abstract

Acetic acid bacteria such as *Acetobacter* spp. produce high concentrations of acetic acid by oxidizing ethanol while reducing molecular oxygen to water on the cell surface. The acetic acid resistance of acetic acid bacteria has been explained as a complex of several mechanisms. The *aarC* gene, encoding succinyl-CoA:acetate CoA transferase, which catalyzes the committed step of acetate metabolism, plays a crucial role in acetic acid resistance in *Acetobacter aceti*. Here, we constructed a mutant of *Acetobacter pasteurianus* strain SKU1108, from which *aarC* and its paralog *aarC2* were deleted (named the ∆∆ strain). The ∆∆ strain failed to grow on a glucose- and glycerol-containing medium in the presence of acetic acid. However, when the ∆∆ strain was cultivated with ethanol, it grew well and produced acetic acid at comparable levels to the parental strain. When ethanol was added to growing cell-based acetic acid resistance experiments, the ∆∆ strain grew even in the presence of acetic acid, suggesting an ethanol-dependent but AarC-independent mechanism of acetic acid resistance. 2-Propanol and lactic acid were also oxidized by the ∆∆ strain and improved acetic acid resistance, though not as much as ethanol. In addition to growing cell assays, we established a resting cell-based cell killing assay with acetic acid. A protonophore, carbonyl cyanide *m*-chlorophenyl hydrazone, inhibited the ethanol-dependent acetic acid resistance of the ∆∆ strain in the cell killing assay. Thus, overall, we find that *A. pasteurianus* has AarC-independent but ethanol-dependent acetic acid resistance that is protonophore-sensitive.

**IMPORTANCE:**

Vinegar is produced by acetic acid fermentation by acetic acid bacteria such as *Komagataeibacter* spp. and *Acetobacter* spp. Resistance to acetic acid is an important feature of these microorganisms. At least two mechanisms have been proposed for acetic acid resistance: acetate metabolism and acetic acid efflux. The gene *aarC* is crucial in the acetate metabolism of *Acetobacter* sp. Here, a mutant derivative of *Acetobacter pasteurianus* strain SKU1108, devoid of *aarC,* failed to grow on acetate. In the absence of ethanol, the mutant was sensitive to acetic acid. However, in the presence of ethanol, it was resistant to acetic acid. These observations suggest a novel mechanism of AarC-independent but ethanol-dependent acetic acid resistance of *A. pasteurianus*. We can now find acetic acid-resistant mechanisms independent of acetate metabolism in growing cell-based experiments, which may promote elucidation of mechanism(s) of acetic acid resistance that involve as-yet-unidentified molecules.

## INTRODUCTION

Vinegar fermentation is carried out in the family Acetobacteraceae by oxidation of ethanol to acetic acid via acetaldehyde as an intermediate compound, coupled with reduction of molecular oxygen to water (1). *Acetobacter pasteurianus* is one of the most commonly observed species of acetic acid bacteria in traditional vinegar fermentation (2). The membrane-bound dehydrogenases alcohol dehydrogenase and aldehyde dehydrogenase oxidize the substrates in the periplasmic space and reduce ubiquinone in the cytoplasmic membrane to ubiquinol (Fig. S1). Ubiquinol is reoxidized by cytochrome *ba*_3_ ubiquinol oxidase, a homolog of bacterial cytochrome *bo*_3_ ubiquinol oxidase, which reduces molecular oxygen to water with consumption of protons (3). By analogy with the *bo*_3_ oxidase, it is postulated that on the oxidation of two molecules of ubiquinol to ubiquinone that releases four protons into the periplasmic space, the *ba*_3_ oxidase reduces one molecule of dioxygen to two molecules of water by consuming four protons in the cytoplasm (4, 5) (Fig. S1). Importantly, the *ba*_3_ oxidase pumps protons from the cytoplasm to the periplasm coupled with the oxidation of ubiquinol (3): four protons are likely pumped per oxidation of two molecules of ubiquinol (6, 7). This process generates an inside-negative proton motive force across the cytoplasmic membrane, which in turn drives ATP synthesis by the H^+^-translocating F_O_F_1_-ATP synthase.

Acetic acid (the protonated form) but not acetate (the deprotonated form) is able to penetrate the phospholipid bilayer at as high a rate as water. Because the acid dissociation constant of acetic acid is 4.7, some portion of acetic acid can enter the cytoplasm even at near neutral pH. If acetic acid reaches the cytoplasm, it is deprotonated because the cytoplasm is neutral, thereby releasing a proton. Such acidification of the cytoplasm is one serious effect of acetic acid on cellular functions. Ishii et al. showed that during acetic acid fermentation, the intracellular pH of the acetic acid bacterium *Komagataeibacter europaeus* strain KGMA0119 was decreased from pH 6.6 to 5.2, while the pH of the culture medium was 2.5 (8). These results clearly indicate that acetic acid produced by fermentation dramatically decreases the intracellular pH. However, acetic acid bacteria must possess a protective system against acidification of the cytoplasm; otherwise, the intracellular pH would be almost equal to that of the culture medium.

Acetic acid resistance is an important feature of *Acetobacter* sp. and involves several mechanisms, such as DNA repair protein UvrA (9), the molecular chaperone GroE (10, 11), acetate metabolism (12, 13), acetic acid efflux (14, 15), specific phospholipid components (16, 17), and pellicle polysaccharide (18). A mutant derivative of *A. aceti* strain no. 1023 that lacked the *aarC* gene (*aarC* stands for acetic acid resistance C) was sensitive to acetic acid and did not assimilate acetate (12). Later, Mullins et al. found that the AarC protein is a succinyl-CoA:acetate CoA transferase (SCAT), and strain no. 1023 lacks a succinyl-CoA synthetase and concluded that acetate metabolism is responsible for acetic acid resistance in *A. aceti* (13).

Two types of acetic acid efflux systems have been proposed for acetic acid resistance in the genus *Acetobacter*. AatA, an ABC transporter, was found in the proteome of *A. aceti* 10-8S2 cells (a derivative of strain no. 1023) grown in the presence of acetic acid, and the *aatA* gene was concluded to be involved in acetic acid resistance (15). In yeast, suppression of intracellular acidification by the H^+^-translocating ATPase Pma1 contributes to acetic acid resistance (19). Proton motive force-dependent acetic acid efflux transporter(s) have also been proposed from a series of biochemical analyses using membrane vesicles of *A. pasteurianus* strain NBRC3283 (then called *A. aceti*) (14). In yeast, Aqr1 (20), Tpo2, and Tpo3 (21, 22), members of the H^+^-antiporter family of the major facilitator superfamily, decrease the intracellular acetic acid level and thus contribute to acetic acid resistance.

However, transporter(s) responsible for proton motive force-dependent acetic acid efflux remain unknown in *Acetobacter* spp. We hypothesized that because the mechanism of acetic acid resistance is complex and composed of several molecular systems, such as degradation, pumping out, and cell surface structure, this complexity may obstruct the identification of relevant transporter(s). Additionally, the action of such efflux systems is not easily found in growing cell-based experiments. Thus, in this study, we attempted to dissect the acetic acid resistance of *A. pasteurianus* strain SKU1108. Strain SKU1108 possesses the *aarC* gene instead of a gene for succinyl-CoA synthetase, and it has a complete tricarboxylic acid (TCA) cycle (23, 24). Because *A. pasteurianus* drives the TCA cycle with AarC by consuming acetate, we attempted to construct a mutant that lacks acetate metabolism by eliminating the *aarC* gene. On eliminating *aarC*, we found a novel acetic acid resistance mechanism in *A. pasteurianus* in growing cell-based assays. This assay system paves the way for the molecular identification of the mechanism. We propose an acetate metabolism-independent but ethanol-dependent acetic acid resistance in *A. pasteurianus*.

## RESULTS

### The *aarC* genes are essential for growth on acetate

**Fig 1:**
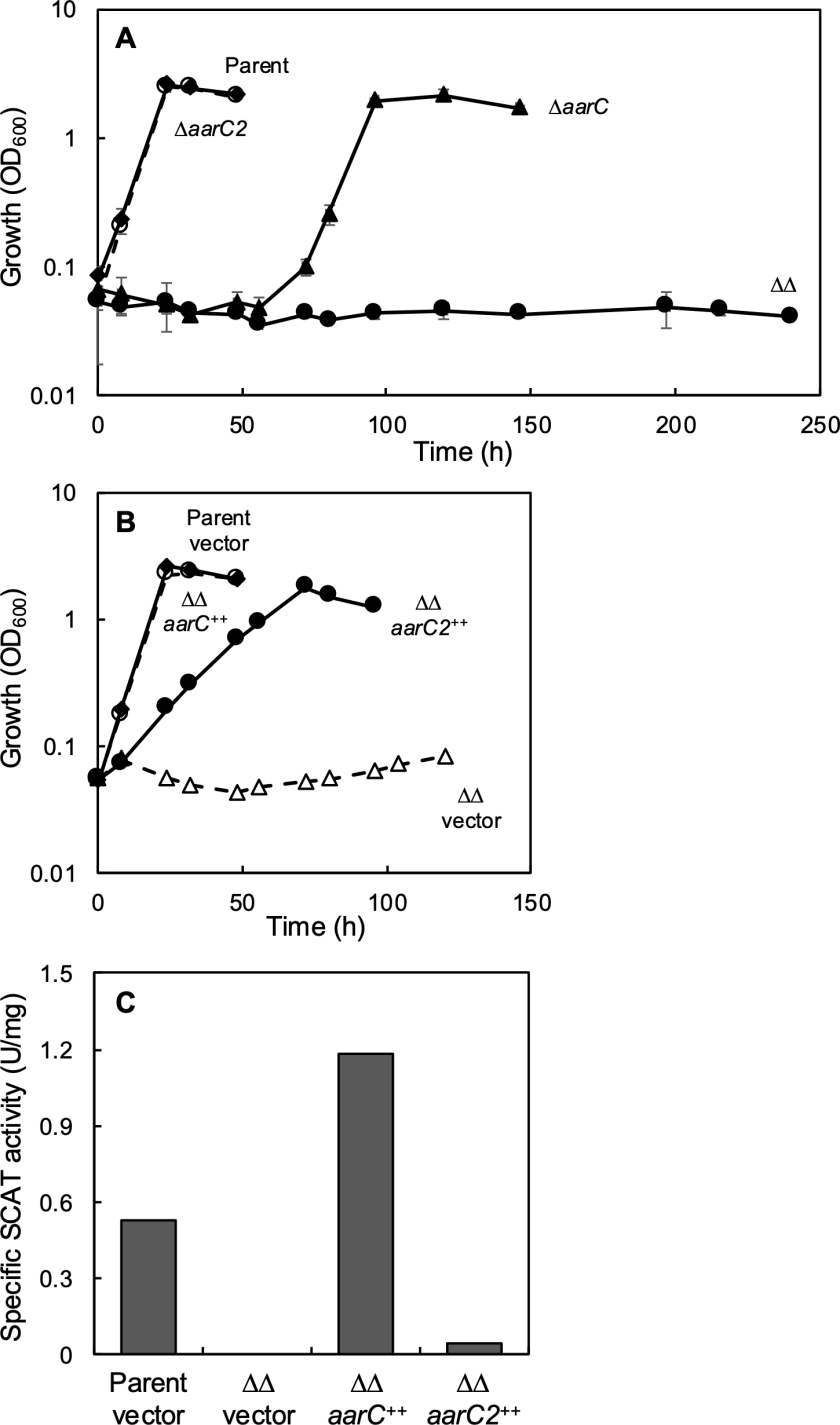
The *aarC* genes are essential for the growth of *A. pasteurianus* on acetate. (**A**) The parental *A. pasteurianus* strain and its derivatives were precultivated in 10 mL of YPGDPi medium at 30°C for 1 d for MS (the parental strain, open circles) and strain MK2 (∆*aarC2*, filled diamonds), and 2 d for strains MK1 (Δ*aarC*, filled triangles) and MK3 (Δ*aarC* Δ*aarC2,* ∆∆, filled circles). One milliliter of preculture was transferred into 100 mL of YPA medium and aerobically shaken at 30°C. Mean values and standard deviations (error bars) are shown from triplicate cultures. (**B**) The parental strain harboring control plasmid (MS/pCM62, open circles), the ∆∆ strain harboring control plasmid (∆∆/pCM62, open triangles), the ∆∆ strain harboring the *aarC*-expression plasmid (∆∆/pMK12, filled diamonds), and the ∆∆ strain harboring the *aarC2*-expression plasmid (∆∆/pMK13, filled circles) were precultivated in 10 mL of YPGPi medium at 30°C for 1 d for MS/pCM62, ∆∆/pMK12 (*aarC*), and ∆∆/pMK13 (*aarC2*), and 2 d for ∆∆/pCM62. The preculture was inoculated into 100 mL of YPA medium and aerobically shaken at 30°C. Mean values and standard deviations (error bars) are shown from triplicate cultures. (**C**) SCAT activity of the MS/pCM62, ∆∆/pCM62, ∆∆/pMK12 (*aarC*), and ∆∆/pMK13 (*aarC2*) strains. Cell-free extracts were prepared from cells grown on YPGPi medium for 29.5 h for MS/pCM62, ∆∆/pMK12 (*aarC*), and ∆∆/pMK13 (*aarC2*), or 36 h for ∆∆/pCM62.

We constructed an *aarC* gene-deletion mutant of *A. pasteurianus* SKU1108 and designated it MK1. The MK1 strain (∆*aarC*) apparently did not grow well on acetate, but it started to grow after approximately 50 h (Fig. 1A). Strain SKU1108 possesses a paralogous gene of *aarC* (tentatively referred to as *aarC2*) in the genome (24); therefore, we postulated that the *aarC2* gene compensated for the loss of *aarC*. The amino acid sequence of AarC shares 65% identity and 94% similarity with AarC2. We thus constructed a ∆*aarC* ∆*aarC2* double-deletion strain (named MK3 or ∆∆), and a ∆*aarC2* single-deletion strain (MK2), to evaluate the role of AarC2 in acetate metabolism. The growth of the parental strain (named MS) and MK2 (∆*aarC2*) was similar. However, the ∆∆ strain did not grow on acetate even after incubation for 10 d (Fig. 1A). Thus, the *aarC2* gene is dispensable if *aarC* works, while inactivation of *aarC* severely affects acetate assimilation even if the strain possesses the *aarC2* gene.

The function of the AarC2 protein was evaluated by complementation tests. We constructed expression plasmids for *aarC* and *aarC2*, respectively, and transformed the *A. pasteurianus* strains with the plasmids as described in the Materials and Methods section. The growth of the parental strain harboring pCM62 (vector control) and the ∆∆ strain harboring pCM62 (Fig. 1B) was similar to the growth of the plasmid-free bacterial strains shown in Fig. 1A, suggesting that the plasmids and antibiotics had little effect on the growth experiments. The ∆∆ strain harboring pMK12 (*aarC*) or pMK13 (*aarC2*) grew better than the vector control strain (∆∆/vector), and pMK12 (*aarC*) complemented the growth of the ∆∆ strain on acetate. The results indicate that the AarC2 protein complements the loss of AarC in acetate metabolism, even though the growth rates of the two recombinant strains differed.

### Growth of the ∆*aarC* ∆*aarC2* strain on glycerol

The gene-deletion mutant derivatives were constructed using ∆P medium, which contains glucose and glycerol. Because the parental strain does not grow well on glucose, we examined the growth behavior of the ∆*aarC* and ∆∆ strains on glycerol. We found that growth of the parental strain elevated the pH of the glycerol medium via an unknown mechanism, and thus, 50 mM potassium dihydrogen phosphate was added to the medium to repress the pH change. Even though the ∆∆ strain has an incomplete TCA cycle, it grew well; nevertheless, the ∆*aarC* and ∆∆ strains showed a longer lag phase and lower cell density at the stationary phase than the parental strain (Fig. S2). The ∆*aarC2* strain grew like the parental strain.

The ∆*aarC* and ∆∆ strains have an incomplete TCA cycle, which may result in a decreased supply of energy. To compensate for an energy decrease in the ∆*aarC* strain, 2-propanol was supplemented as an oxidation substrate. Because strain SKU1108 possesses PQQ4 dehydrogenase (25) in its genome, 2-propanol is oxidized to acetone via a PQQ4 dehydrogenase-dependent cell surface oxidation system in the periplasmic space (Fig. S1 for reference). On the addition of 2-propanol to YPGPi medium, the cell density of the ∆*aarC* strain at the stationary phase increased to that of the parental strain (Fig. S3). However, a longer lag phase was still observed in the initial stage of growth. Nevertheless, we suggest that an increased supply of energy improved the cell density in the stationary phase of the ∆*aarC* strain grown on glycerol.

Loss of *aarC* presumably induces decreased intracellular levels of C4-dicarboxylates. Thus, C4-dicarboxylates (succinate, fumarate, or L-malate) were added to YPGPi medium. However, the ∆*aarC* strain still showed the extended lag phase in early growth, even in the presence of both C4-dicarboxylate and 2-propanol.

We anticipated that the Δ*aarC* strain would accumulate acetate when grown on glycerol because acetate is a substrate of AarC. However, by using high-performance liquid chromatography (HPLC) systems, we could not detect acetate or any metabolites in the glycerol medium on which the parental and Δ*aarC* cells grew. Thus, glycerol metabolism seems different from that of lactate and pyruvate, and we cannot currently determine how glycerol is metabolized by *A. pasteurianus* strain SKU1108.

### SCAT activity of AarC2

Here, SCAT activity of the *A. pasteurianus* strains was measured. The cells were cultivated on YPGPi medium because glycerol medium produces the highest cell density (as described above). Most SCAT activity was lost in the ∆*aarC* strain (Fig. S2): the activity was approximately 1% of that in the parental strain, almost at the detection limit. The ∆*aarC2* strain showed SCAT activity like that of the parental strain. In the ∆∆ strain, SCAT activity was below the detection limit, suggesting that only the *aarC* and *aarC2* genes are responsible for SCAT activity.

We also measured the SCAT activity of recombinant strains, that is, the ∆∆ strain harboring pMK12 (*aarC*) or pMK13 (*aarC2*) grown on glycerol (Fig. 1C). The parental strain harboring the control plasmid and the ∆∆ strain harboring pKM12 (*aarC*) showed high SCAT activity. The SCAT activity of the ∆∆ strain harboring pKM13 (*aarC2*) was about 1/10th of that in the parental strain. We do not have any definitive explanation for the low SCAT activity in the ∆∆/pKM13 (*aarC2*) strain, though it could result from a low enzyme expression level or different substrate specificity. We suggest that the lower SCAT activity of the ∆∆/pKM13 (*aarC2*) strain is one of the reasons for the slow growth of this strain on acetate (Fig. 1B).

### The ∆*aarC* ∆*aarC2* strain is sensitive to acetic acid, yet it produces a high level of acetic acid

**Fig 2:**
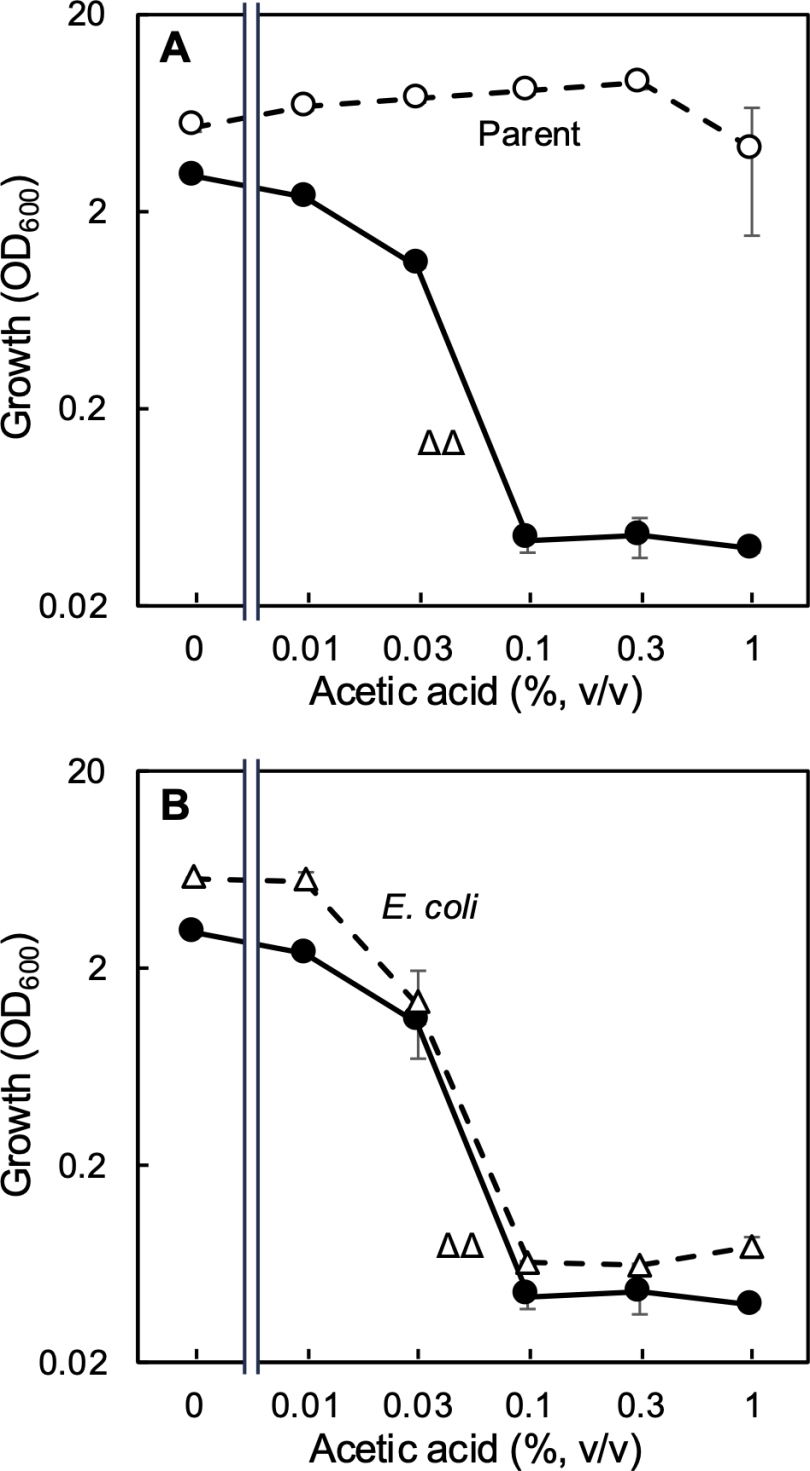
The Δ*aarC* ∆*aarC2* derivative of *A. pasteurianus* SKU1108 is sensitive to acetic acid. (**A**) *A. pasteurianus* strains MS (parental, open circles) and MK3 (Δ*aarC* ∆*aarC2*, closed circles, ∆∆) were precultivated in ∆P medium and transferred to YPGD medium supplemented with 0.01%, 0.03%, 0.1%, 0.3%, or 1% (vol/vol) acetic acid. The main cultivation was conducted at 30°C for 48 h with shaking at 150 rpm. (**B**) Comparison of acetic acid sensitivity of the ∆∆ strain (closed circles, the same data as in panel **A**) with that of *Escherichia coli* (open triangles). *E. coli* strain BW25113 was precultivated in modified Luria–Bertani medium and transferred to the same medium supplemented with 0.01%, 0.03%, 0.1%, 0.3%, or 1% (vol/vol) acetic acid. The main cultivation was conducted at 30°C for 24 h with shaking at 150 rpm. Mean values and standard deviations (error bars) are shown from triplicate cultures.

The *aarC* gene was originally found to be a factor involved in acetic acid resistance (12, 26). To confirm that our ∆*aarC* ∆*aarC2* strain is indeed sensitive to acetic acid, we designed an acetic acid resistance test based on growing cells using YPGD medium (including glucose and glycerol without buffer, see the Materials and Methods section) containing 0.01%, 0.03%, 0.1%, 0.3%, or 1.0% (vol/vol) acetic acid. The parental and ∆∆ cells were precultivated in ∆P medium and transferred to the acetic acid resistance test. A similar acetic acid resistance test was conducted for *Escherichia coli* strain BW25113. For *E. coli*, modified Luria–Bertani medium was used for preculture and the acetic acid resistance test contained the same concentrations of acetic acid as those that we used here for *A. pasteurianus* (Fig. 2). The *A. pasteurianus* parental strain MS grew even in the presence of 1% acetic acid, whereas the ∆∆ strain did not grow in the presence of ≥0.1% acetic acid. *E. coli*, as a comparison, also failed to grow in the presence of ≥0.1% acetic acid, indicating that the acetic acid resistance of the ∆∆ strain was decreased to the same level as that of *E. coli* by the loss of AarC.

**Fig 3:**
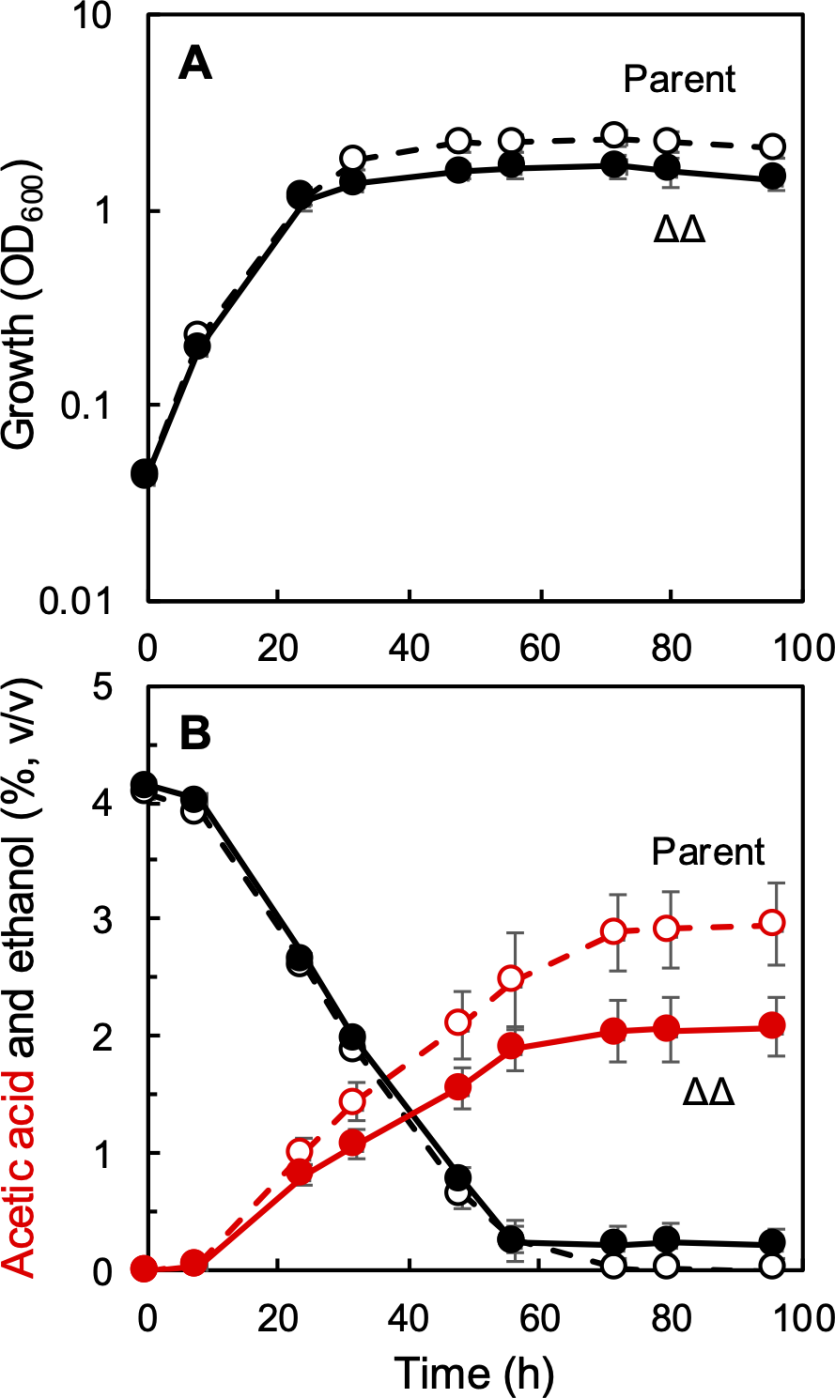
Acetic acid production by the MK3 (Δ*aarC* Δ*aarC2*) strain of *A. pasteurianus*. The *A. pasteurianus* MS (parental, open symbols) and MK3 (Δ*aarC* Δ*aarC2*, filled symbols, ∆∆) strains were precultivated in ∆P medium and transferred to YPGD medium supplemented with 4% (vol/vol) ethanol. The culture was incubated at 30°C with shaking at 200 rpm. (**A**) Growth and (**B**) acetic acid (red) and ethanol (black) levels. Mean values and standard deviations (error bars) are shown from triplicate cultures.

The acetic acid fermentation ability of the ∆∆ strain was evaluated. Because the ∆∆ strain possesses ethanol oxidation ability, we hypothesized that it may produce some acetic acid, but that the growth of this strain would be inhibited by the produced acetic acid. Ethanol was added to YPGD (glucose and glycerol) medium at 4% (vol/vol), and the growth, ethanol consumption, and acetic acid production of the ∆∆ strain were compared with those of the parental strain (Fig. 3). To our surprise, the ∆∆ strain was still able to grow well, and it produced 2.1% (vol/vol) acetic acid, comparable to the parental strain (3.0% [vol/vol]). The ∆∆ strain showed a lower final cell density than the parental strain, but it consumed ethanol similarly to the parental strain. The acetic acid production ability of the ∆∆ strain contradicts the results of the acetic acid resistance test described above, in which the ∆∆ strain failed to grow even in the presence of 0.1% acetic acid (Fig. 2). These results suggest that the acetic acid resistance of *A. pasteurianus* may be enhanced by ethanol.

### AarC-independent but ethanol-dependent acetic acid resistance

**Fig 4:**
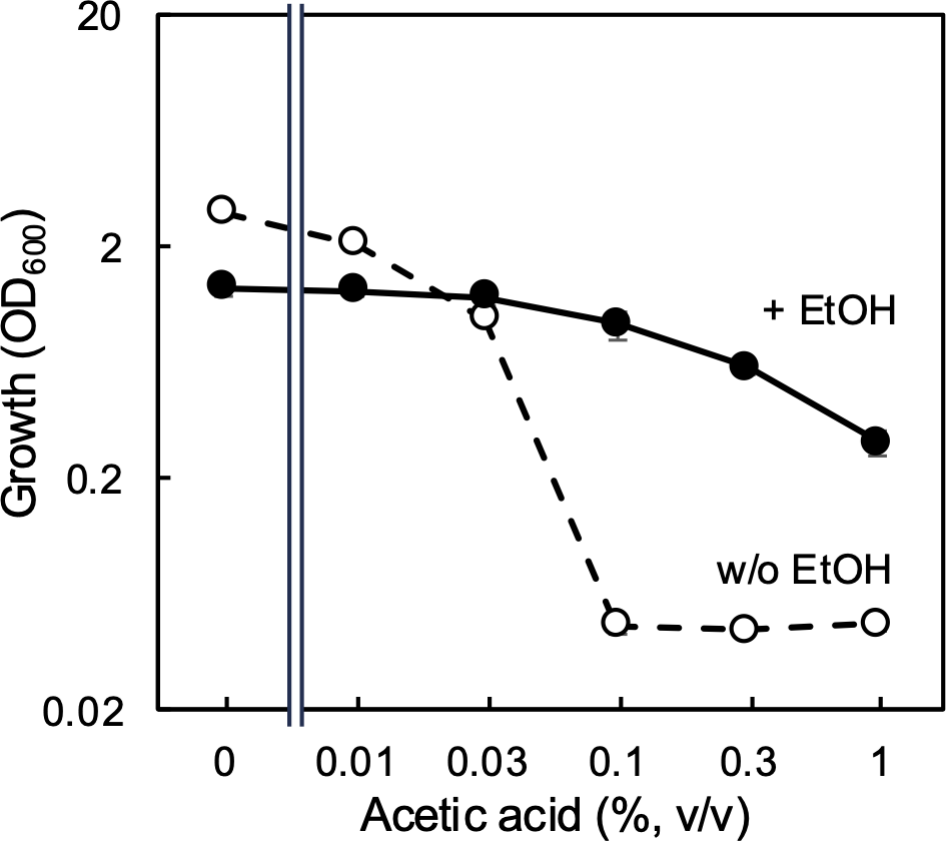
Effect of ethanol on acetic acid resistance of the MK3 (∆∆) strain. An acetic acid resistance test (as described in the legend of Fig. 2) was conducted for the ∆∆ strain in the presence (filled circles) or absence (open circles) of 1% (vol/vol) ethanol. Mean values and standard deviations (error bars) are shown from triplicate cultures.

We next evaluated the effect of ethanol on the acetic acid resistance of the ∆∆ strain. The acetic acid resistance test described above was conducted again, but ethanol was supplemented at 1% (vol/vol) (Fig. 4). The ∆∆ strain was able to grow even in the presence of 1.0% acetic acid when ethanol was also present. These results strongly suggest an AarC-independent acetic acid resistance system driven by ethanol in *A. pasteurianus* SKU1108.

**Fig 5:**
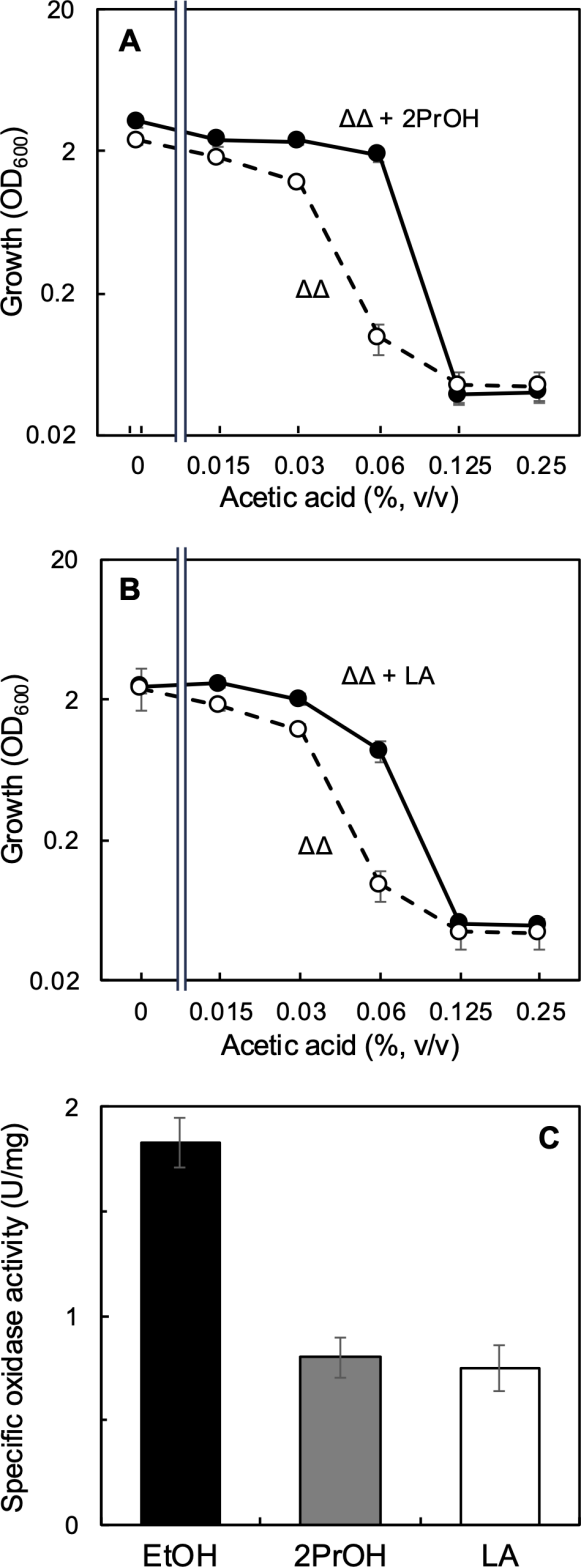
Acetic acid resistance of the MK3 (∆∆) strain in the presence of oxidation substrates. (**A**) Effect of 2-propanol on the growth of the MK3 (Δ*aarC* Δ*aarC2*) strain of *A. pasteurianus* in the presence of acetic acid. An acetic acid resistance test was conducted for the ∆∆ strain in the presence (filled circles) or absence (open circles) of 2% (vol/vol) 2-propanol as described in the legend of Fig. 2. Mean values and standard deviations (error bars) are shown from triplicate cultures. (**B**) Effect of lactic acid on the growth of the MK3 strain in the presence of acetic acid. An acetic acid resistance test was conducted for the ∆∆ strain in the presence (filled circles) or absence (open circles) of 0.1% (vol/vol) racemic lactic acid as described in the legend of Fig. 2. Mean values and standard deviations (error bars) are shown from triplicate cultures. (**C**) Oxidase activity of the MK3 strain. MK3 cells were cultivated in ΔP medium at 30°C with shaking at 200 rpm for 24 h. Then, the cells were harvested and washed. The oxygen consumption rate of cell suspension in the presence of 1% (vol/vol) ethanol (EtOH, black), 2% (vol/vol) 2-propanol (2PrOH, gray), or 0.1% (vol/vol) lactic acid (LA, white) was measured in 100 mM MES–KOH (pH 6.0) using an oxygen electrode. Mean values and standard deviations (error bars) are shown from triplicate assays.

We examined whether other oxidation substrates, such as 2-propanol and lactic acid, would confer similar resistance to acetic acid as that conferred by ethanol. Because ethanol is the substrate of a cell-surface ethanol oxidation system for acetic acid fermentation that produces proton motive force, we anticipated that the operation of cell-surface oxidation is key to the AarC-independent acetic acid resistance. We thus examined 2% (vol/vol) 2-propanol instead of ethanol in the acetic acid resistance test with a range of acetic acid concentrations (Fig. 5A). 2-Propanol clearly improved the growth of the ∆∆ strain in the presence of acetic acid, particularly at 0.06% (vol/vol) acetic acid. However, the acetic acid resistance enabled by 2-propanol was low compared with that enabled by ethanol. Another oxidation substrate, lactic acid, also conferred resistance to 0.06% acetic acid (Fig. 5B) because sodium lactate would elevate the pH of the medium, lactic acid (which decreases the pH) was used in this experiment.

Oxidase activities of the ∆∆ cells with ethanol, 2-propanol, and lactic acid were determined by measuring the oxygen consumption rate. The oxidase activity was highest with ethanol; the activities with 2-propanol and lactic acid were approximately half of that with ethanol and comparable to each other (Fig. 5C). Considering that the acetic acid resistance test used lactic acid rather than sodium lactate, lactic acid was also used in the oxidase assay: the pH decreased to 5.1 from 6.0. These results suggest that high oxidation activity on the cell surface is important for the AarC-independent acetic acid resistance of *A. pasteurianus* SKU1108.

### Ethanol-dependent acetic acid resistance is sensitive to the protonophore carbonyl cyanide *m*-chlorophenyl hydrazone

The cell-surface oxidation systems driven by ethanol, 2-propanol, and lactic acid produce proton motive force, which, in turn, drives F_O_F_1_-ATPase to produce ATP. Here, we examined the effect of the protonophore carbonyl cyanide *m*-chlorophenyl hydrazone (CCCP) on the AarC-independent but ethanol-dependent acetic acid resistance of *A. pasteurianus* SKU1108 because CCCP eliminates the proton motive force. However, we needed a different experimental setup to evaluate acetic acid resistance independent of cell growth because the acetic acid resistance test (Fig. 2) depends on cell growth that would be inhibited by CCCP. We thus established the “cell killing assay” described in the Materials and Methods section, in which the conditions are similar to growth conditions, but in which the cells die (killed by acetic acid).

**Fig 6:**
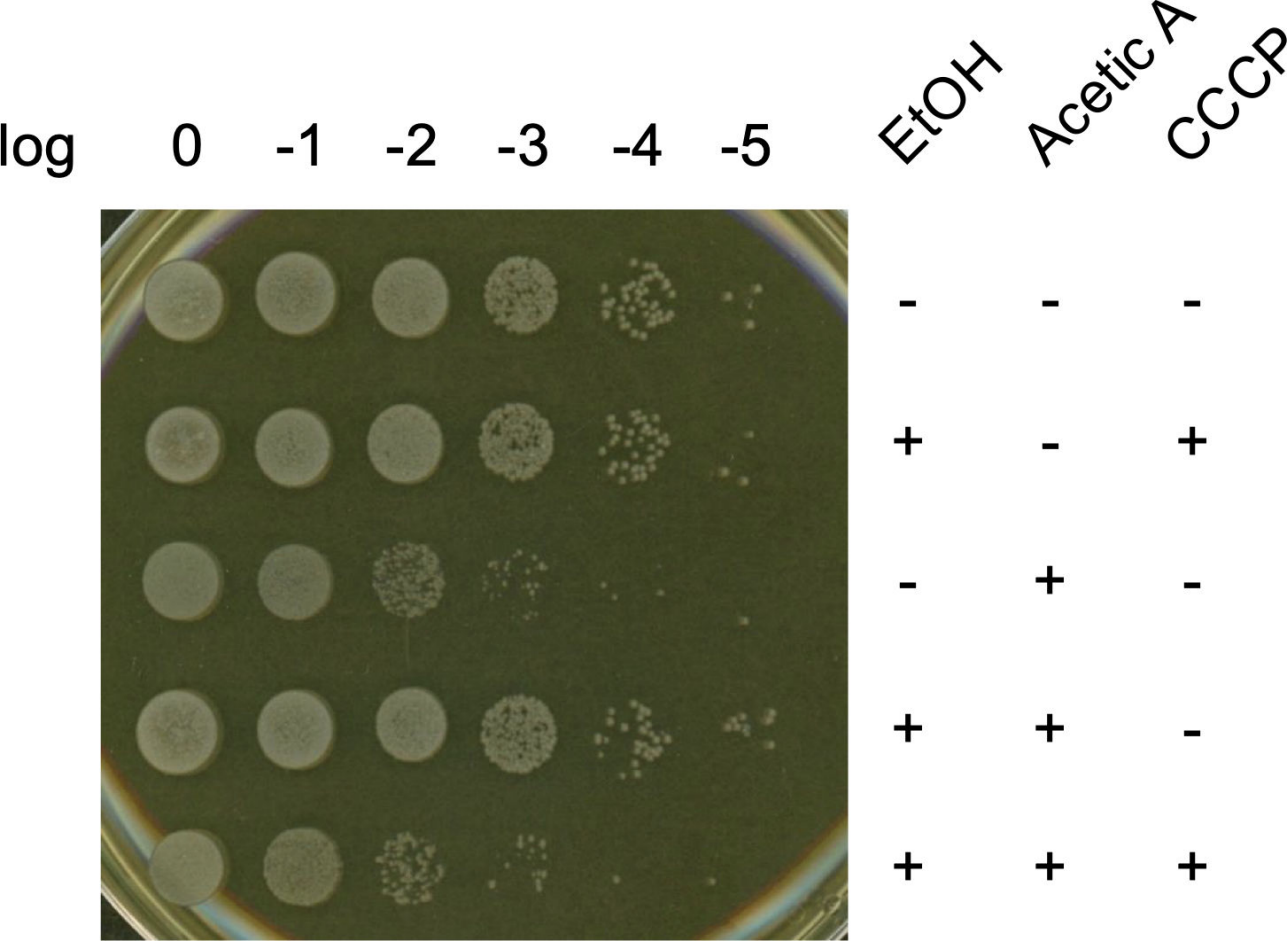
Acetic acid kills *A. pasteurianus* cells; that effect is alleviated by ethanol, but the protective effect is eliminated by the protonophore CCCP. The *A. pasteurianus* MK3 (Δ*aarC* ∆*aarC2*, ∆∆) strain was cultivated in ΔP medium at 30°C with shaking at 200 rpm for 20 h. Then, the cells were harvested and washed twice with 150 mM KCl. The cell suspension was incubated in 150 mM KCl at 30°C with shaking at 160 rpm for 30 min with (+) or without (−) the following components: 1% (vol/vol) ethanol (EtOH), 7.6% (vol/vol) acetic acid (pH 3.7, Acetic A), and 200 µM CCCP. After a 30-min incubation, dipotassium hydrogen phosphate was added to neutralize acetic acid. The cell suspension was serially diluted 10-fold with 150 mM KCl. Seven microliters of each dilute cell suspension was spotted onto ΔP-agar and incubated at 30°C for 2 d.

Acetic acid of 7.6% (vol/vol) killed *A. pasteurianus* ∆∆ cells in the cell killing assay at pH 3.7, decreasing the viable cells by a factor of 10^2^ (Fig. 6). Ethanol and CCCP did not affect the number of viable cells in this assay (Fig. 6, second line). However, the damaging effect of acetic acid was completely alleviated by the addition of 1% (vol/vol) ethanol, showing that the ethanol-dependent acetic acid resistance can still be observed in an assay system not based on cell growth, that is, a resting cell-based assay. These results were reproduced when the parental strain, in which AarC is present, was used in the cell killing assay (Fig. S4), suggesting that the damaging effect of acetic acid in this experiment is not alleviated by the action of AarC. The number of viable *A. pasteurianus* ∆∆ cells was decreased by the addition of the protonophore CCCP to the condition containing ethanol and acetic acid (Fig. 6, the bottom line), indicating that elimination of the proton motive force inhibits the ethanol-dependent acetic acid resistance of *A. pasteurianus*. Therefore, it can be suggested that molecular event(s) driven by proton motive force and/or ATP are involved in the ethanol-dependent acetic acid resistance because proton motive force is connected to ATP synthesis. Another possible explanation is the redistribution of protons that were pumped out by the cellular system, such as via cell-surface oxidation, into the cytoplasm on the addition of CCCP, decreasing the intracellular pH to a lethal level.

The ABC transporter AatA was found to be involved in acetic acid resistance in *A. aceti* strain 10-8S2 (15). We thus evaluated the possibility that the AatA homolog in *A. pasteurianus* SKU1108 contributes to the ethanol-dependent acetic acid resistance. Strain OM4 (∆*aarC* ∆*aarC2* ∆*aatA*) still showed acetic acid resistance in the growing cell-based acetic acid resistance test (Fig. S5), suggesting that the ABC transporter AatA plays only a limited part, if any, in the AarC-independent but ethanol-dependent acetic acid resistance of *A. pasteurianus* SKU1108.

## DISCUSSION

Through a series of biochemical analyses using membrane vesicles, we previously proposed that a proton motive force-dependent efflux system for acetic acid is one of the major strategies for acetic acid resistance in *Acetobacter* sp (14). However, the protein(s) or gene(s) involved in such an efflux system have remained unclear. We anticipated that difficulties in identifying molecule(s) functioning in acetic acid efflux would arise from the presence of other mechanisms for acetic acid resistance, that is, AarC, in the cells. That is, even though we postulated a proton motive force-dependent efflux system for acetic acid from assays using cell membranes, it has proved difficult to find the system by using growing cell methods. In this study, we attempted to dissect the acetic acid resistance systems in *Acetobacter* sp., that is, we constructed a mutant derivative of *A. pasteurianus* SKU1108 without the two *aarC* genes: *aarC* and *aarC2*. As we describe in the Results section, the double-deletion (∆∆) strain showed ethanol-dependent acetic acid resistance in growing cell-based assays (Fig. 4). This acetic acid resistance was reproducible on agar medium: the ∆∆ cells formed colonies on YPGD-agar containing acetic acid, only when ethanol was supplemented (unpublished observation). These growing cell-based assays provide us an outlook for understanding the molecular mechanism of acetic acid resistance. We are currently attempting to screen acetic acid-sensitive mutants from the ∆∆ strain generated through random mutagenesis to identify the gene(s) responsible for the resistance mechanism.

**Fig 7:**
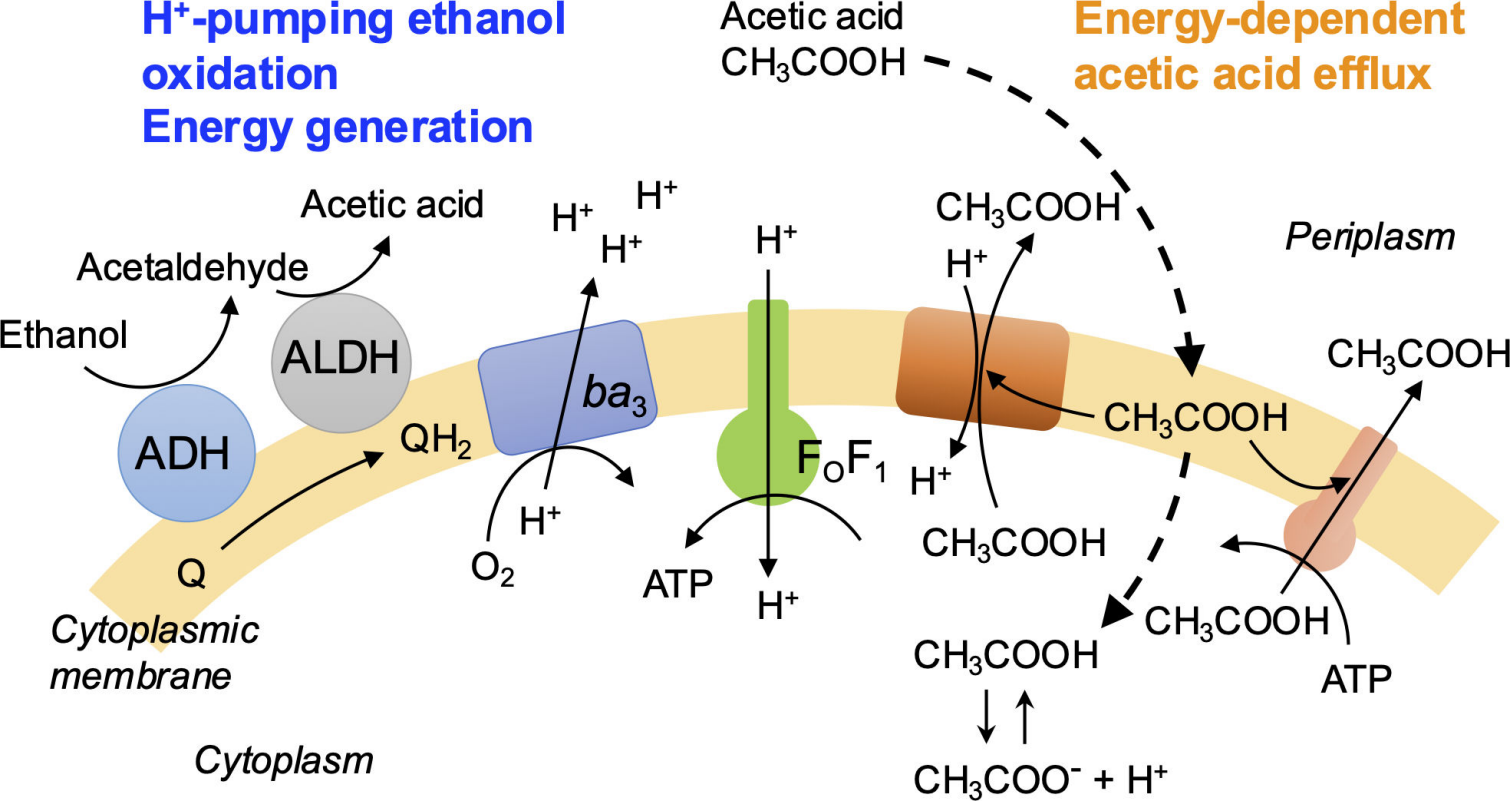
Hypothetical model for acetate metabolism-independent but ethanol-dependent acetic acid resistance of *A. pasteurianus*. Ethanol is oxidized to acetic acid by membrane-bound alcohol dehydrogenase (ADH) and membrane-bound aldehyde dehydrogenase (ALDH), and molecular oxygen is reduced to water by cytochrome *ba*_3_ ubiquinol oxidase (*ba*_3_) with the consumption of protons. The *ba*_3_ oxidase also pumps protons, which generates the proton motive force. F_O_F_1_-ATP synthase (F_O_F_1_) produces ATP by consuming the proton motive force, transporting protons into the cytoplasm. Acetic acid (protonated form) penetrates the cytoplasmic membrane (thick dashed arrow). Acetic acid in the cytoplasm changes into acetate (the deprotonated form) and releases protons. Decreasing the intracellular pH would inhibit basic cellular functions such as metabolism and protein synthesis. Acetic acid (the protonated form) would also accumulate in the cytoplasmic membrane, distorting the membrane integrity. We suggest that H^+^-pumping by the ethanol oxidation system (left side) alleviates intracellular acidification, which is one of the mechanisms for acetate metabolism-independent acetic acid resistance of *A. pasteurianus* strain SKU1108. Another mechanism may be acetic acid efflux by one or more as-yet-unidentified proton motive force- or ATP-dependent acetic acid transporter(s) (right side, tangerine and salmon molecules respectively) to decrease the acetic acid level in the cytoplasmic membrane.

There are several candidates for molecules involved in protonophore-sensitive acetic acid resistance in *A. pasteurianus* (Fig. 7). Because the toxicity of acetic acid would arise from acidification of the cytoplasm, decreasing the proton concentration in the cytoplasm is one mechanism to resist acetic acid. In this context, an ATP-dependent H^+^-pump, such as an H^+^-pumping P-type ATPase homologous to Pma1 of *Saccharomyces cerevisiae* (19), or the reverse reaction of F_O_F_1_-ATP synthase, may be candidates for an ATP-dependent transporter, as found in lactic acid bacteria (27). One gene for a P-type ATPase homolog (APT_01976) is present in the *A. pasteurianus* SKU1108 genome. However, if one proton is pumped by the consumption of one ATP molecule, the P-type ATPase homolog would not alleviate intracellular acidification because the F_O_F_1_-ATP synthase intakes approximately three protons for the synthesis of one ATP molecule.

H^+^-pumping by the cell surface oxidation system is a possible mechanism of acetic acid resistance (Fig. 7; Fig. S1), which would be more effective than H^+^-pumping by the reverse reaction of F_O_F_1_-ATP synthase. In *E. coli*, activation of the respiratory chain is proposed to suppress intracellular acidification by weak acids (28, 29), and such a mechanism should be considered in *Acetobacter*. In *Acetobacter*, when one molecule of ethanol is oxidized to acetic acid, one molecule of molecular oxygen is reduced to water with consumption of four protons, and cytochrome *ba*_3_ ubiquinol oxidase likely pumps four protons out of the cytoplasm: thus, oxidation of one molecule of ethanol to acetic acid may alleviate acidification (penetration of the cytoplasmic membrane) by eight molecules of acetic acid. The genome of strain SKU1108 possesses genes for a cytochrome *bd*-type ubiquinol oxidase (APT_01544-01542) and those for a cyanide-insensitive ubiquinol oxidase (APT_02214-02212), neither of which pump protons (30–32). We are attempting to use ubiquinol oxidases that do not pump protons to examine the hypothesis that the cell surface oxidation system is responsible for acetic acid resistance in *A. pasteurianus*.

We suggest that the cell-surface ethanol oxidation system alleviates intracellular acidification by acetic acid in *A. pasteurianus*, but it does not fully account for the acetic acid resistance of this species. A strain of *Gluconobacter oxydans* (then called *G. suboxydans*) was shown to possess a high ethanol oxidation activity similar to that of an *A. pasteurianus* (then called *A. aceti*) strain (33), but the acetic acid resistance of *Gluconobacter* spp. is generally lower than that of *A. pasteurianus*, suggesting the presence of additional acetic acid-resistant machinery in *A. pasteurianus*. A major facilitator superfamily transporter (the tangerine-colored protein in Fig. 7) is a possible acetic acid-resistant system, as suggested by our group in 2005 (14), but this mechanism would not effectively resist acidification by acetic acid if the coupling ion is H^+^ (which would acidify the cytoplasm upon acetic acid efflux). Acetic acid has a partition coefficient between octanol and water similar to that of ethanol. Particularly at high concentrations, it is conceivable that acetic acid may have some adverse effects on the cytoplasmic membrane like those of hydrophobic substances (34). Considering only the suppression of intracellular acidification as described above, the proton motive force-dependent acetic acid efflux system in *A. pasteurianus* SKU1108 seems to contribute only a limited part of the overall resistance to acetic acid. However, if it also facilitates acetic acid efflux from the cytoplasmic membrane, it likely contributes to acetic acid resistance by mitigating the adverse effects of acetic acid on the membrane (Fig. 7). This would presumably also be true for an ATP-dependent acetic acid efflux system. Because the AatA ABC transporter appears not to work in acetic acid resistance in cells of strain SKU1108 (Fig. S5), another ATP-dependent acetic acid efflux system may be responsible for such a mechanism (Fig. 7).

## MATERIALS AND METHODS

### Chemicals

Acetyl-CoA trilithium salt and succinyl-CoA sodium salt were from Fujifilm Wako (Osaka, Japan) and Cayman Chemical (Ann Arbor, MI), respectively. Yeast extract was supplied by Oriental Yeast (Tokyo, Japan). Hipolypepton was from Shiotani M.S. (Amagasaki, Japan). All other chemicals used in this study were commercial products.

### Bacterial strains, plasmids, and culture conditions

The bacterial strains and plasmids used in this study are listed in Table 1. *A. pasteurianus* strain SKU1108 has been deposited in the NBRC (http://www.nite.go.jp/en/nbrc/index.html) with accession number NBRC 101655. The MS strain, a smooth-surfaced colony derivative of *A. pasteurianus* strain SKU1108, was used in this study as the parental strain. *A. pasteurianus* strains were grown on ∆P medium (10 g yeast extract, 10 g Hipolypepton, 5 g D-glucose, 20 g glycerol per liter); YPGD medium (5 g yeast extract, 5 g Hipolypepton, 5 g glycerol, 5 g D-glucose per liter); YPGDPi medium (5 g yeast extract, 5 g Hipolypepton, 5 g glycerol, 5 g D-glucose, 6.8 g potassium dihydrogen phosphate per liter, pH 6.5 [adjusted for the complete medium with KOH]); YPGPi medium (5 g yeast extract, 5 g Hipolypepton, 5 g glycerol, 6.8 g potassium dihydrogen phosphate per liter); and YPA medium (5 g yeast extract, 5 g Hipolypepton, 5 g acetic acid per liter, pH 5.0 [adjusted for the complete medium with NaOH]). *E. coli* strain DH5α was used for plasmid construction (35). *E. coli* strain HB101 harboring pRK2013 was used for triparental mating (36). *E. coli* strain BW25113 was used in acetic acid resistance tests (37). *E. coli* strains were grown on modified Luria–Bertani medium (10 g Hipolypepton, 5 g yeast extract, 5 g NaCl per liter, pH 7.0 [adjusted with NaOH]). Tetracycline and kanamycin were used at final concentrations of 10 and 50 µg mL^−1^, respectively, for *E. coli*, and 50 and 50 µg mL^−1^, respectively, for *A. pasteurianus*.

**TABLE 1:** *Acetobacter pasteurianus* strains and plasmids used in this study

Strains and plasmids	Relevant characteristics	Source or reference
*Acetobacter pasteurianus* strains		
SKU1108	Wild type	(38)
MS	Smooth-surfaced colony derivative of SKU1108	This study
MK1	MS Δ*aarC*	This study
MK2	MS Δ*aarC2*	This study
MK3	MS ∆*aarC* Δ*aarC2*	This study
OM4	MS ∆*aarC* Δ*aarC2* Δ*aatA*	This study
Plasmids		
pKOS6b	Suicide vector, *mob, lacZα, codAB*, Km^R^	(39)
pRK2013	Plasmid mediates plasmid transfer, Km^R^	(36)
pCM62	Broad-host-range vector, *mob, lacZα*, Tc^R^	(40)
pMK07	pKOS6b, a 1.6 kb ∆*aarC* allele	This study
pMK11	pKOS6b, a 1.6 kb ∆*aarC2* allele	This study
pMK12	pCM62, a 2.0 kb *aarC* fragment	This study
pMK13	pCM62, a 2.6 kb *aarC2* fragment	This study
pAN107	pKOS6b, a 2.2 kb ∆*aatA* allele	This study

### Determination of acetic acid

Culture was taken and centrifuged at 10,000 × *g* for 5 min at 4°C, and the supernatant was filtered through a 0.45 µm membrane (ANPEL Laboratory Technologies, Shanghai, China) before HPLC analysis (Shimadzu, Kyoto, Japan). Samples were run on an ion exclusion column (RSpak KC-811, 8.0 mm [inside diameter] by 300 mm long; Shodex, Showa Denko KK, Kawasaki, Japan) at 60°C using 0.1% (wt/vol) phosphoric acid as the mobile phase at a flow rate of 0.7 mL min^−1^. Acetic acid was detected using a diode array detector at 210 nm.

### Construction of plasmids

The oligonucleotides for use as DNA primers are listed in Table 2. The *aarC* (*APT_01020*) gene of *A. pasteurianus* SKU1108 was amplified by using Herculase II DNA polymerase (Stratagene, CA) using primer pair 1108-D-aarC-5-Hin (+) and 1108-D-aarC-3-Xba (−). The PCR product of approximately 2.8 kb was digested with *Pst*I and *Xba*I to obtain a 0.8 kb DNA fragment containing the downstream region of *aarC*. The upstream region of the *aarC* gene was amplified from genomic DNA of strain SKU1108 using Herculase II fusion DNA polymerase and primer pair 1108-D-aarC-5-Hin (+) and 1108-D-aarC-5-Pst (−). The PCR product of approximately 0.8 kb was digested with *Hin*dIII and *Pst*I to obtain a 0.8 kb DNA fragment containing the upstream region of *aarC*. The two 0.8 kb DNA fragments were ligated with pKOS6b (39) treated with *Hin*dIII and *Xba*I to construct pMK07, a deletion plasmid for the *aarC* gene.

**TABLE 2:** Oligonucleotides used in this study

Oligonucleotide	Sequence (5′–3′)	Objective
1108-D-aarC-5-Hin(+)	aagcttccagatgaggggtgg	∆*aarC*
1108-D-aarC-5-Pst(−)	ctgcaggctgatctggtatttc	∆*aarC*
1108-D-aarC-3-Xba(−)	tctagattgtttgcacaccggg	∆*aarC*
1108-ex-aarC-HinApa(+)	aagcttgggcccacaatgcggagtcc	∆*aarC*^+^
1108-ex-aarC-KpnSal(−)	gtcgacggtacctcgctttagaagatgc	∆*aarC*^+^
1108-D-aarC2-5-Sph(+)	gcatgcggtcttctaaacgatcc	∆*aarC2*
1108-D-aarC2-5-Pst(−)	ctgcagttcttcggccgtcatgacac	∆*aarC2*
1108-D-aarC2-3-RI(−)	gaattcctcccgcaccagtgaaag	∆*aarC2*
1108-DaatA-5-EcoHin(+)	gaattcaagcttgtccagagcacgcacaattg	∆*aatA*
1108-DaatA-5-Eco52(−)	cggccgcgccatccagaagtggg	∆*aatA*
1108-DaatA-Sph(−)	gcatgcgtgtatcccaatggcgg	∆*aatA*

Plasmid pKM11, a gene-deletion plasmid for the *aarC2* (*APT_01656*) gene of *A. pasteurianus* SKU1108, was constructed by following similar procedures to those used for *aarC* and oligonucleotide primers 1108-D-aarC2-5-Sph(+), 1108-D-aarC2-5-Pst (−), and 1108-D-aarC2-3-RI(−). Plasmid pAN107, a gene-deletion plasmid for the *aatA* (*APT_01245*) gene of *A. pasteurianus* SKU1108, was constructed by following similar procedures and oligonucleotide primers 1108-DaatA-5-EcoHin(+), 1108-DaatA-5-Eco52(−), and 1108-DaatA-Sph(−).

The *aarC* gene of *A. pasteurianus* SKU1108 was amplified using Herculase II DNA polymerase and primer pair 1108-ex-aarC-HinApa(+) and 1108-ex-aarC-KpnSal(−). The PCR product of approximately 2.0 kb was digested with *Hin*dIII and *Kpn*I to obtain a 2.0 kb DNA fragment containing the whole *aarC* gene. The DNA fragment was ligated with pCM62 (40) treated with *Hin*dIII and *Kpn*I to construct pMK12, an *aarC* expression plasmid.

Plasmid pKM13, an expression plasmid for *aarC2*, was constructed by following similar procedures to those for *aarC* using oligonucleotide primers 1108-D-aarC2-5-Sph(+) and 1108-D-aarC2-3-RI(−). *Hin*dIII and *Eco*RI were used for ligation of the PCR product with pCM62 to construct pMK13, which carries *aarC2* and a gene for APT_01657 (a hypothetical protein) in the opposite direction.

### Construction of bacterial strains

*A. pasteurianus* strain SKU1108 was transformed by a triparental mating method as described previously (41). Gene-deletion derivatives of *A. pasteurianus* were constructed using suicide plasmids as described previously (41). Kanamycin-resistant transformants were examined to determine if they were recombinant strains that carried a deletion of the gene of interest by PCR. Fluorocytosine (60 µg mL^−1^; Tokyo Chemical Industry, Tokyo, Japan) was used for counterselection of second recombinants: fluorocytosine-resistant strains were examined for whether they had lost kanamycin resistance. Second recombinant strains were examined to determine whether they were gene-deletion mutants or the wild type by checking the length of the gene of interest by PCR, as described previously (41).

### Preparation of cell suspensions

Preculture of strain MK3 (∆*aarC* ∆*aarC2*) grown in ΔP medium was inoculated into 100 mL ΔP medium in a 500 mL Erlenmeyer flask and incubated at 30°C for 24 h with shaking at 200 rpm. The cells were collected by centrifugation at 8,000 × *g* for 10 min at 4°C and washed with 10 mM 2-(*N*-morpholino)ethanesulfonic acid (MES)–KOH (pH 6.0) containing 1 mM CaCl_2_, twice. The cells were resuspended in 10 mM MES–KOH (pH 6.0) containing 1 mM CaCl_2_ in a ratio of 4 mL per gram of wet cells and used as cell suspensions. Protein content was determined by the Bradford method (42), and bovine serum albumin was used as the standard.

### Preparation of cell-free extracts

Preculture of *A. pasteurianus* strains grown in YPGDPi medium was inoculated into 100 mL YPGPi medium in a 500 mL Erlenmeyer flask and incubated at 30°C for the periods stated with shaking at 200 rpm. The cultivation periods of the MK1 (∆*aarC*) and MK3 (∆*aarC* ∆*aarC2*) strains were longer than those of the MS (parental) and MK2 (∆*aarC2*) strains. The cells were collected by centrifugation at 8,000 × *g* for 10 min at 4°C and washed with 25 mM 4-(2-hydroxyethyl)−1-piperazineethanesulfonic acid (HEPES; pH 8.0) containing 10 mM NaCl, twice. The cells were resuspended in 25 mM HEPES (pH 8.0) containing 10 mM NaCl in a ratio of 4 mL per gram wet cell weight. Then, the cell suspension was passed through a French cell press (American Instrument, Co.) at 1,000 kg cm^−2^, twice. Unbroken cells were removed by centrifugation at 8,000 × *g* at 4°C for 10 min. The supernatant was used as cell-free extract. Protein content was determined by the Bradford method (42), and bovine serum albumin was used as the standard.

### Cell killing assay

*A. pasteurianus* cells were precultivated in ∆P medium at 30°C for 24 h. The preculture was transferred to 100 mL of YPGD medium containing 4% (vol/vol) ethanol in a 500 mL Erlenmeyer flask at a cell density such that OD_600_ = 0.02. The main culture was incubated at 30°C with shaking at 200 rpm for 20 h. The cells were collected by centrifugation at 10,000 × *g* for 10 min at 20°C, washed with 150 mM KCl twice, and resuspended in 150 mM KCl to OD_600_ = 12. The cells were incubated in 7.6% (vol/vol) acetic acid, of which the pH had been adjusted to 3.7 with NaOH, in the presence or absence of 1% (vol/vol) ethanol and 200 µM CCCP dissolved in dimethyl sulfoxide. The density of the cell suspension was such that OD_600_ = 1.2; 10 mL of the cell suspension in a 100 mL Erlenmeyer flask was incubated at 30°C with shaking at 150 rpm for 30 min. Then, 12 mL of 1 M dipotassium hydrogen phosphate was added to the cell suspension to neutralize the acetic acid. The cell suspension was serially diluted 10-fold with 150 mM KCl, and aliquots of the diluted cell suspension were put on ∆P-agar followed by incubation at 30°C for 2 d to check the number of viable cells.

### Enzyme assays

#### AarC activity

AarC assay was conducted at 25°C by following the method described by Mullins et al. (13). The mixture contained 0.2 mM succinyl-CoA sodium salt, 350 mM potassium acetate, 100 mM potassium chloride, 50 mM potassium phosphate (pH 8.0), and cell-free extract as the enzyme. After 0, 7, 15, and 30 min, an aliquot (100 µL) of the reaction mixture was mixed with 400 µL of 6.25% trichloroacetic acid, then centrifuged at 10,000 × *g* for 5 min at 4°C. The supernatant was filtered through a 0.45 µm Millex filter (Millipore, Burlington, MA) before HPLC analysis. Acetyl-CoA was determined by using a Shimadzu HPLC system (Kyoto, Japan) as described previously (13) with a Symmetry C18 column (4.6 mm × 75 mm, 3.5 µm; Waters, Milford, MA) at 35°C. The conditions for the HPLC analysis followed the method described previously (13). Acetyl-CoA was detected by using a diode array detector at 260 nm. Acetyl-CoA trilithium salt was used to generate a calibration curve for acetyl-CoA. One unit of AarC activity was defined as 1 µmol of acetyl-CoA produced per min.

#### Oxidase activity

Oxidase activity was measured using a Clark-type oxygen electrode (YSI model 5300, Yellow Spring Instrument, Yellow Springs, OH, USA) at 25°C. The electrode was calibrated by using air-saturated 100 mM K^+^-MES (pH 6.0), assuming the concentration of molecular oxygen to be 249 µM (43). Sodium dithionite was used to reduce molecular oxygen completely for calibration. Reaction mixture (total volume 1.5 mL) contained intact cells, 100 mM K^+^-MES (pH 6.0), and the indicated concentrations of substrate. One unit was defined as 1 µmol of half a molecule of oxygen (equivalent to one oxygen atom) consumed per min.

## Data Availability

*Acetobacter pasteurianus* strain SKU1108 has been deposited in the NBRC (http://www.nite.go.jp/en/nbrc/index.html) with accession number NBRC 101655. The complete genome sequence has been deposited in DDBJ/EMBL/GenBank under accession no. AP014881 to AP014885. The data that support the findings of this study are available from the corresponding author upon reasonable request.

## SUPPLEMENTAL MATERIAL

Supplemental figures aem.02034-25-s0001.pdf Figures S1 to S5.

ASM does not own the copyrights to Supplemental Material that may be linked to, or accessed through, an article. The authors have granted ASM a non-exclusive, world-wide license to publish the Supplemental Material files. Please contact the corresponding author directly for reuse.
