# Supplementary material for: Evidence for ethanol-dependent acetic acid resistance in Acetobacter pasteurianus strain SKU1108
Source: Appl Environ Microbiol. 2025 Nov 26;91(12):e02034-25. doi: 10.1128/aem.02034-25 (PMC12724360; doi:10.1128/aem.02034-25)
Supplement: Supplemental figures — Figures S1 to S5. [file aem.02034-25-s0001.pdf]

Supporting Information

Evidence for ethanol-dependent acetic acid resistance in *Acetobacter pasteurianus* strain SKU1108

Akari Narimatsu[1,‡], Kaho Murakami[2,‡], Naoya Kataoka[2,3,4], Riku Yamashita[2], Kazunobu Matsushita[2,3,4], Minenosuke Matsutani[5], Uraiwan Tippayasak[6], Gunjana Theeragool[1,6], and Toshiharu Yakushi[1,2,3,4,#]

[1]Joint Degree Program of Kasetsart University and Yamaguchi University, Graduate School of Science and Technology for Innovation, Yamaguchi University, Yamaguchi 753-8515, Japan; [2]Division of Agricultural Science, Graduate School of Science and Technology for Innovation, Yamaguchi University, Yamaguchi 753-8515, Japan; [3]Department of Biological Chemistry, Faculty of Agriculture, Yamaguchi University, Yamaguchi 753-8515, Japan; [4]Research Center for Thermotolerant Microbial Resources, Yamaguchi University, Yamaguchi 753-8515, Japan; [5]Department of Food, Aroma and Cosmetic Chemistry, Faculty of Bioindustry, Tokyo University of Agriculture, Hokkaido 099-2493, Japan; [6]Department of Microbiology, Faculty of Science, Kasetsart University, Bangkok 10900, Thailand.

[#]Corresponding author.
Toshiharu Yakushi

ORCID, 0000-0003-2279-8968
Graduate School of Science and Technology for Innovation, Yamaguchi University, 1677-1 Yoshida, Yamaguchi 753-8515, Japan
Tel: +81-83-933-5858; Fax: +81-83-933-5820; Email: juji@yamaguchi-u.ac.jp

[‡]These authors contributed equally to this work.

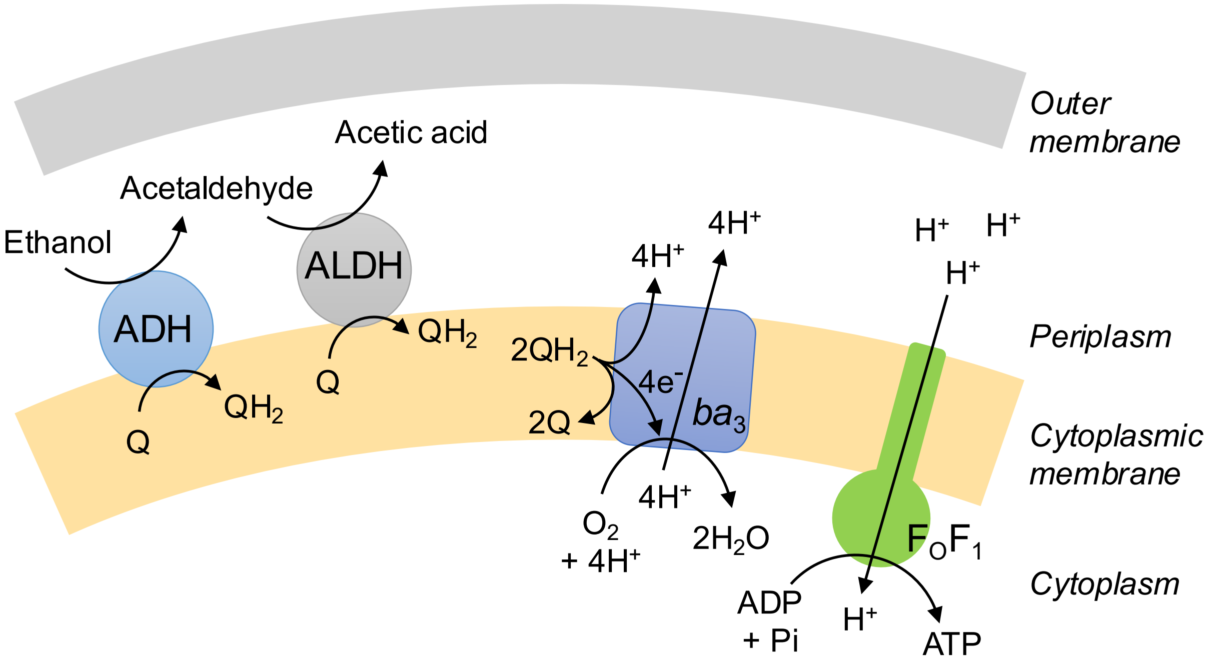

Fig. S1. Narimatsu et al.

Fig. S1. Model of cell-surface ethanol oxidation system in acetic acid bacteria. Ethanol is oxidized into acetic acid in the periplasmic space by membrane-bound alcohol dehydrogenase (ADH) and membrane-bound aldehyde dehydrogenase (ALDH). The two dehydrogenases reduce ubiquinone (Q) in the cytoplasmic membrane to ubiquinol (QH_2). Cytochrome ba_3 ubiquinol oxidase (ba_3) oxidizes QH_2 to Q, where the protons are released to the periplasm, and reduces molecular oxygen with consumption of protons in the cytoplasm. The ba_3 oxidase pumps protons ($2H^+/QH_2$), coupled with the chemical reaction, generating proton motive force. When the ba_3 oxidase oxidizes two molecules of QH_2 derived from oxidation of one molecule of ethanol to acetic acid, it reduces one molecule of O_2 and consumes four protons, coupling with pumping four protons out. F_OF_1-ATP synthase (F_OF_1) synthesizes ATP from ADP and phosphate by consuming proton motive force, translocating protons as the coupling ions.

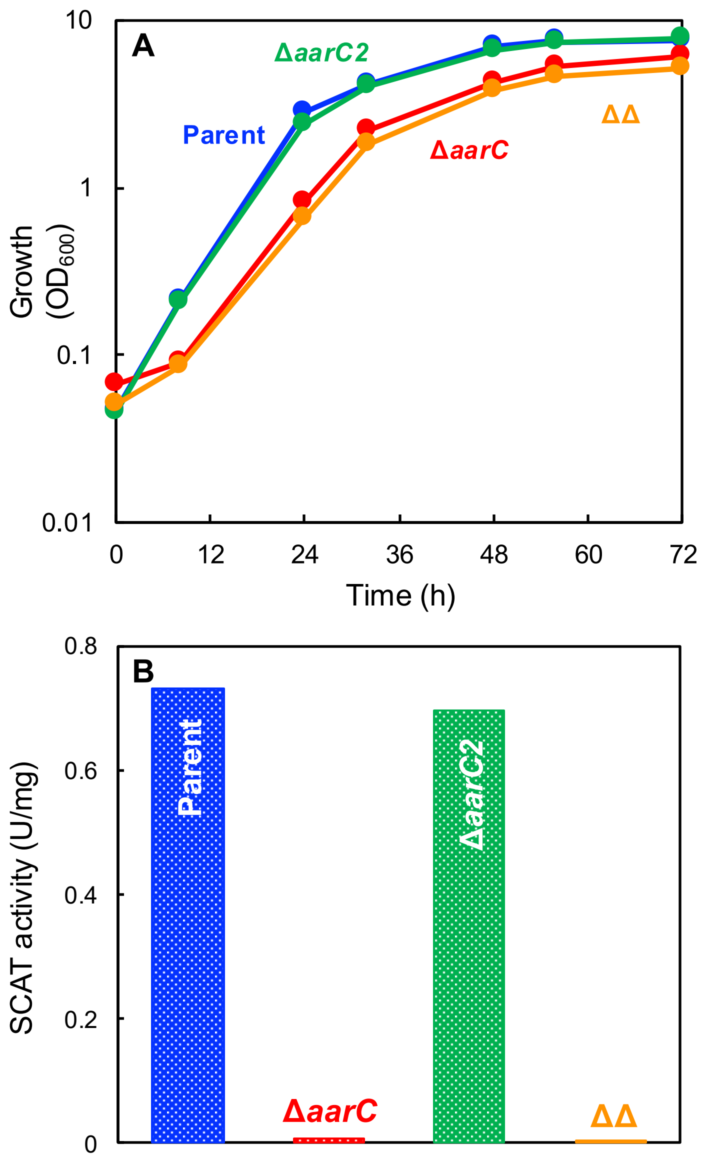

Fig. S2. Narimatsu et al.

Fig. S2. Growth of the MS, Δ*aarC*, Δ*aarC2* and Δ*aarC* Δ*aarC2* strains on YPGPi medium and succinyl-CoA:acetate CoA transferase (SCAT) activity.
A: Strains MS (parental, blue), MK2 (Δ*aarC2*, green), MK1 (Δ*aarC*, red) and MK3 (Δ*aarC* Δ*aarC2*, ΔΔ, orange) were precultivated in 10 mL of YPGDPi medium at 30°C for 1 d for the parental and Δ*aarC2* strains and 2 d for the Δ*aarC* and ΔΔ strains. The preculture was inoculated into 100 mL of YPGPi medium and aerobically shaken at 30°C for 72 h. B: SCAT activity of the MS, Δ*aarC*, Δ*aarC2* and ΔΔ strains. Cell-free extracts were prepared from cells grown in 100 mL of YPGPi medium in 500-mL flasks for 30 or 46 h. Mean values and standard deviations (error bars) are shown from triplicate enzyme assays. The activity of strain ΔΔ was below the detection limit.

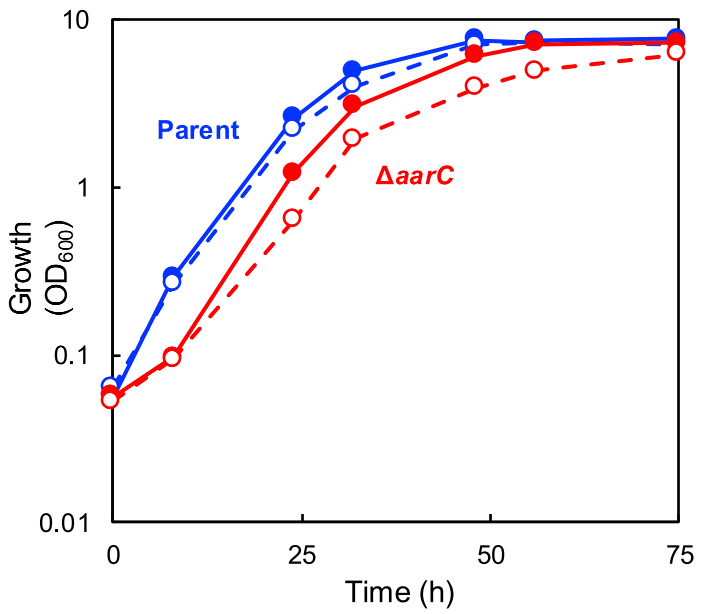

Fig. S3. Narimatsu et al.

Fig. S3. 2-Propanol promotes the growth of the Δ*aarC* strain on glycerol.
Strain MS (parental, blue) and MK1 (Δ*aarC*, red) cells were precultivated in 10 mL of YPGPi medium at 30°C for 1 d for the parental strain and 2 d for the Δ*aarC* strain. The preculture was inoculated into 100 mL of YPGPi medium supplemented with (filled symbols) or without (open symbols) 0.1% (v/v) 2-propanol. The main culture was conducted by shaking at 30°C.

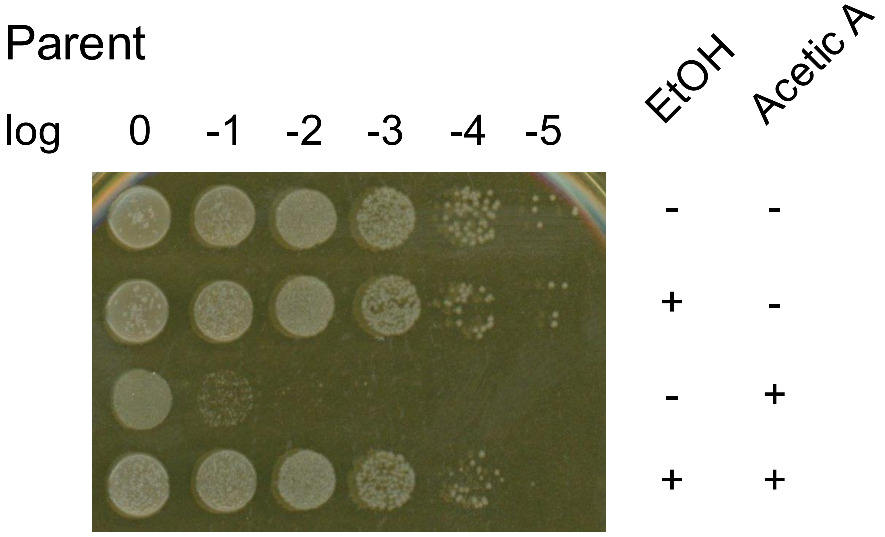

Fig. S4. Narimatsu et al.

Fig. S4. Acetic acid kills the parental *A. pasteurianus* strain MS, but ethanol alleviates acetic acid sensitivity.
The cell killing assay was conducted with the MS (parental) strain as described in the legend of Fig. 6.

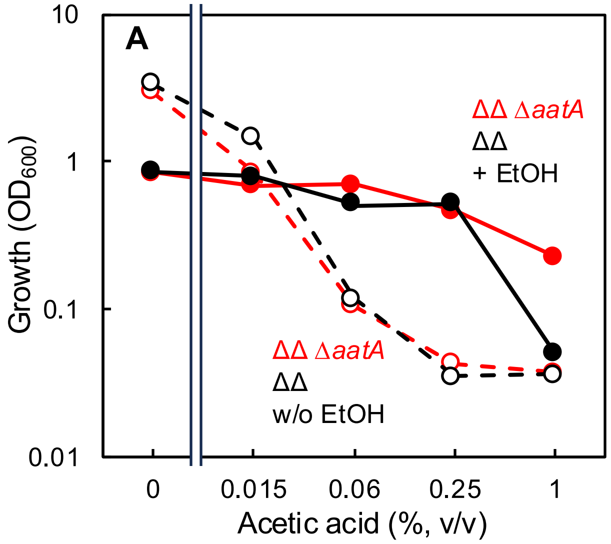

Fig. S5. Narimatsu et al.

Fig. S5. The ABC transporter AatA is not involved in AarC-independent acetic acid resistance.
Acetic acid resistance tests were conducted with strains MK3 (ΔΔ, black) and OM4 (Δ*aarC* Δ*aarC2* Δ*aatA*, red, indicated as "ΔΔ Δ*aatA*" in the figure) in the presence (filled circles) or absence (open circles) of 1% (v/v) ethanol as described in the legend of Fig. 2.
